# Firth logistic regression for rare variant association tests

**DOI:** 10.3389/fgene.2014.00187

**Published:** 2014-06-19

**Authors:** Xuefeng Wang

**Affiliations:** Program in Public Health, Departments of Preventive Medicine, Biomedical Informatics, and Applied Mathematics and Statistics, Stony Brook UniversityStony Brook, NY, USA

**Keywords:** rare variants, association test, firth logistic regression, penalized likelihood, GEE

In association tests of sites with low minor allele frequency or count, it is known that single-variant tests are impractical to use because the results from which will be either underpowered or unreliable. Joint analyses by pooling or “collapsing” multiple variants based on annotated gene group information are thus more preferred in rare variant association tests. However, the issue remains in a genome-wide association scan because there is always a portion of regions containing less number of variant sites. Moreover, most current exome or genome sequencing association studies are still limited to small sample sizes. Standard testing methods that rely on the asymptotic theories will also not preserve the type I error rate. These factors together will distort the final genome-wide quantile–quantile plot of the testing *p*-values. A penalized likelihood based method called Firth logistic regression method may provide a simple yet effective solution. It is easier to implement and less computational intensive than alternative approaches such as permutation or bootstrapping, and worthy of more attention in association studies of sequencing data.

The basic idea of the firth logistic regression is to introduce a more effective score function by adding an term that counteracts the first-order term from the asymptotic expansion of the bias of the maximum likelihood estimation—and the term will goes to zero as the sample size increases (Firth, 1993; Heinze and Schemper, 2002). For generalized linear models with canonical links such as in logistic regression, Firth’s approach is equivalent to penalizing the likelihood by the Jeffreys invariant prior. The attraction of this method is that it provides bias-reduction for small sample size as well as yields finite and consistent estimates even in case of separation. In a binary response model, separation issue occurs when one variant is associated with only one type of outcome, e.g., when all individuals who carry a particular variant (although rare) are diagnosed with the disease. The phenomenon is more commonly seen in rare variants studies, especially when a recessive model is assumed. These variants are undoubtedly important but will not be detected by standard statistical packages as they often report large *p*-values (and exceptionally larger standard errors)—sometimes even without a warning message. Although approaches like Fisher's exact test and exact logistic regression can be used to handle the separation problem, their use become problematic when there are continuous covariates need to be considered. The implementation of firth logistic regression is fairly easy as it is now available in many standard packages (such as R package “logistf”). In a recent work, Ma et al. (2013) performed simulations to compare different methods for the rare variant association test over varied designs and gave promising results. They showed that the firth-regression-based joint analysis of the individual-level data controls type I error well for both balanced and unbalanced studies, and which is more powerful than score test based meta-analysis.

However, methods and software are yet to be developed to handle analyses with family or related samples. Two options are available to handle familial correlations. One is to incorporate Firth correction into the structure of conditional logistic regression (CLR) (Heinze and Puhr, 2010). The other possibility (may be easier) is based on generalized estimation equations (GEE). A simple approximation can be readily applied in practice by modifying standard GEE through the following two steps. First, get the leverage values (diagonal of hat-matrix) from a GEE analysis with independence working correlation; Then add half a leverage to each response before rerunning GEE based on a chosen working correlation matrix. Such procedure will not completely remove the first-order term of the bias, but will adjust toward that direction. This approximation will guarantee finite estimates when separation occurs. Further investigation is, however, needed to test the robustness of the suggested methods to factors such as ascertainment and pedigree structures.

## Conflict of interest statement

The author declares that the research was conducted in the absence of any commercial or financial relationships that could be construed as a potential conflict of interest.

## References

[B1] Firth D. (1993). Bias reduction of maximum likelihood estimates. Biometrika 80, 27–38 10.1093/biomet/80.1.27

[B2] Heinze G. Puhr R. (2010). Bias-reduced and separatio-proof conditional logistic regression with small or sparse data sets. Stat. Med. 29, 770–777 10.1002/sim.3794 20213709

[B3] Heinze G. Schemper M. (2002). A solution to the problem of separation in logistic regression. Stat. Med. 21, 2409–2419 10.1002/sim.1047 12210625

[B4] Ma C. Blackwell T. Boehnke M. Scott L. J. (2013). Recommended joint and meta-analysis strategies for case-control association testing of single low-count variants. Genet. Epidemiol. 37, 539–550 10.1002/gepi.21742 23788246 PMC4049324

